# Frontal fibrosing alopecia: report of four sisters^☆^

**DOI:** 10.1016/j.abd.2022.02.009

**Published:** 2023-07-17

**Authors:** Jéssica Vianna Starek, Thaís Petry Raszl, Samar Mohamad El Harati Kaddourah

**Affiliations:** Department of Dermatology, Complexo Hospitalar Padre Bento, Guarulhos, SP, Brazil; Department of Dermatology, General Dermatology Outpatient Clinic, Trichoses, Complexo Hospitalar Padre Bento, Guarulhos, SP, Brazil

Dear Editor,

Frontal fibrosing alopecia (FFA) belongs to the group of lymphocytic cicatricial alopecias and was first described in 1994 by Kossard.^1^ Clinically, retraction of the frontotemporal hair implantation line is observed, often associated with loss of eyebrows and, in some cases, loss of hair from other parts of the body.^2^

There is a predilection for the female sex and Caucasian individuals, particularly in the postmenopausal period.^3^ The first reports of FFA in individuals from the same family appeared in 2010 when the occurrence of the disease was described in two sisters.^4^ The etiopathogenesis of FFA is still unknown, but the genetic predisposition has been reinforced by its association with some class I human leukocyte antigen (HLA) alleles. As the incidence has been increasing over the years, it is postulated that current environmental triggers may act on a genetic predisposition, driving the th1/JAK-STAT inflammation profile in FFA.^2^

This family consists of five black sisters, aged between 56 and 66 years, all of which have already gone through menopause. The youngest of them came to the dermatology outpatient clinic complaining of thinning hair and, after being asked about her family history, she reported that she had sisters with a similar condition, and thus, all of them were invited to come for an appointment to be evaluated. After the clinical examination, it was found that four of them were affected by FFA (Figure 1, Figure 2), and the diagnosis was also confirmed by anatomopathological examination (Fig. 3). They are from and currently live in the urban area of São Paulo; they lived together until adolescence, and all of them have undergone hair straightening procedures since childhood. They were born to the same parents, who are already deceased and were evaluated through photographs, which showed the mother’s hair without alterations, whereas the father had signs of androgenetic alopecia. Only one of the sisters, aged 63, had a normal scalp.

**Figure 1:**
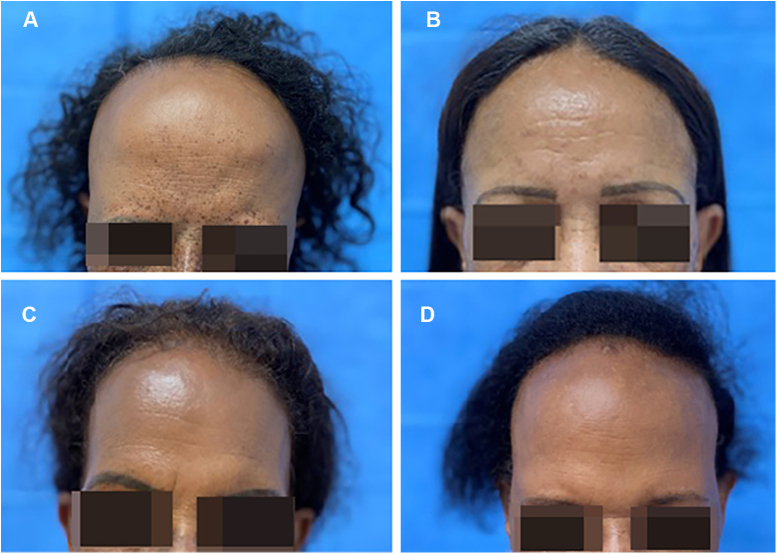
**(A‒D)** Clinical presentation of the four sisters affected by frontal fibrosing alopecia (FFA)

**Figure 2:**
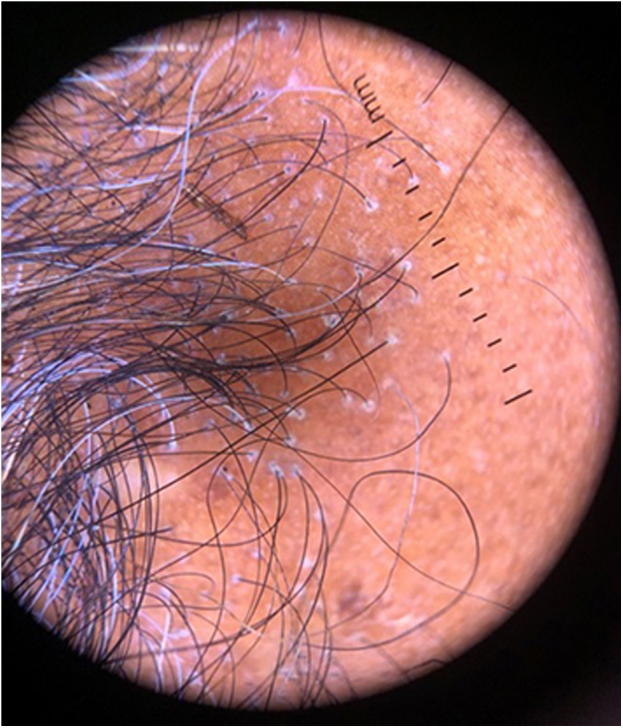
Trichoscopy of one of the patients corroborating the diagnosis. Peri-hair shaft desquamation, follicular units with only one emerging hair shaft, absence of follicular orifices, absence of vellus hairs and ivory-white background with erythema in the area of fibrosis. All patients had a similar pattern

**Figure 3:**
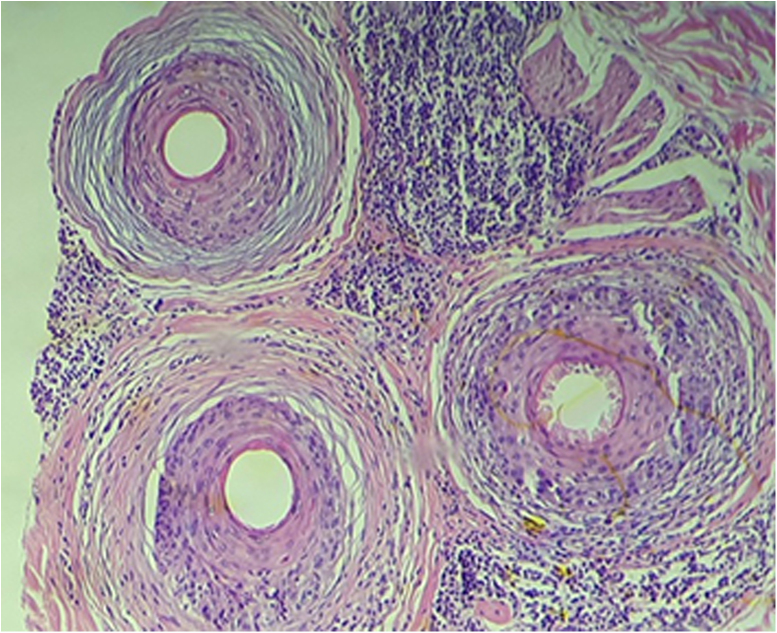
Histopathology of the scalp, isthmus level; (Hematoxylin & eosin, ×100). Cicatricial alopecia with perifollicular concentric fibrosis, lymphocytic inflammation in and around the follicular epithelium and presence of apoptosis. The infundibulum and bulb region did not show any inflammation

The affected patients showed the same clinical pattern, classified as the linear pattern or type I, when there is linear retraction of the hair implantation line. The age at the disease onset ranged from 40 to 62 years. The youngest sister had the most advanced condition, being the only one with disease onset before menopause. She had a history of using regular sunscreen protection since adolescence, longer than the other sisters, who reported irregular use for a few years. Three of them lacked eyebrow hairs, and all had facial lesions suggestive of lichen planus pigmentosus, which is often associated with FFA, especially in patients with a higher phototype.^5^ Among the comorbidities most often related to FFA, one had arthritis (unspecified) and another had hyperthyroidism. The clinical data are detailed in Table 1.

**Table 1:** Clinical data of four sisters with frontal fibrosing alopecia

Patient	Age (years)	Phototype	Age at symptoms onset (years)	Clinical subtype	Lichen planus pigmentosus	Papules on the face	Eyebrow alopecia	Menopause (years)	Comorbidities	Hair treatments
1	56	V	40	I	Present	Absent	Present	55	SAH, unspecified arthritis	Previous hair straightening (adolescence)
2	61	V	59	I	Present	Absent	Present	50	DM, Dyslipidemia, Bipolarity, Smoking	Hair straightening and dying
3	64	V	60	I	Present	Absent	Present	48	DM, Glaucoma	Hair straightening and dying
4	66	V	62	I	Present	Absent	Absent	50	SAH, Hyperthyroidism, Glaucoma	Hair straightening and dying

DM, Diabetes mellitus; SAH, Systemic arterial hypertension.

Since its description, FFA has been reported mainly in Caucasian individuals. Due to the lack of data in the literature, it cannot be stated with conviction whether the prevalence in African descendants is actually lower or if this population has been less studied when compared to Caucasians.^5^

FFA is a relatively recent disease and its prevalence has been increasing in recent years. Since African descent ethnicity and the genetic component have been described in a minority of cases, the authors highlight the relevance of reporting this family series.

## Financial support

None declared.

## Authors’ contributions

Jéssica Vianna Starek: Approval of the final version of the manuscript; drafting and editing of the manuscript; critical review of the literature; critical review of the manuscript.

Thaís Petry Raszl: Approval of the final version of the manuscript; drafting and editing of the manuscript; critical review of the literature; critical review of the manuscript.

Samar Mohamad El Harati Kaddourah: Approval of the final version of the manuscript; critical review of the manuscript.

## Conflicts of interest

None declared.
